# *Daphnia* stress response to environmental concentrations of chloramphenicol—multi-omics approach

**DOI:** 10.1007/s11356-024-35045-4

**Published:** 2024-09-25

**Authors:** Malgorzata Grzesiuk, Marta Grabska, Agata Malinowska, Bianka Świderska, Elzbieta Grzesiuk, Damian Garbicz, Adrian Gorecki

**Affiliations:** 1 Department of Biochemistry and Microbiology, Institute of Biology; Warsaw, University of Life Sciences (SGGW), Warsaw, Poland; 2 Institute of Biochemistry and Biophysics, Polish Academy of Sciences, Pawińskiego 5a, 02-106 Warsaw, Poland; 3 Maria Sklodowska-Curie National Research Institute of Oncology, Roentgena 5, Warsaw, Poland

**Keywords:** Life history, Antibiotics, Microbiome, Proteome

## Abstract

Commonly used medicines, when discarded or improperly disposed of, are known to contaminate freshwater ecosystems. Pharmaceuticals can be toxic and mutagenic, and can modify freshwater organisms, even at environmentally relevant concentrations. Chloramphenicol (CAP) is an antibiotic banned in Europe. However, it is still found in surface waters around the world. The aim of this study was to evaluate the impact of chloramphenicol contamination in freshwater on the model organism *Daphnia magna*. Specific life history parameters, proteome, and host-associated microbiome of four *D. magna* clones were analyzed during a three-generation exposure to CAP at environmental concentrations (32 ng L^−1^). In the first generation, no statistically significant CAP effect at the individual level was detected. After three generations, exposed animals were smaller at first reproduction and on average produced fewer offspring. The differences in *D. magna*’s life history after CAP treatment were in accordance with proteome changes. *D. magna*’s response to CAP presence indicates the high stress that the tested organisms are under, e.g., male production, upregulation of ubiquitin-conjugating enzyme E2 and calcium-binding protein, and downregulation of glutathione transferase. The CAP-exposed *D. magna* proteome profile confirms that CAP, being reactive oxygen species (ROS)-inducing compounds, contributes to structural changes in mitochondria. Microbiome analysis showed a significant difference in the Shannon index between control and CAP-exposed animals, the latter having a more diverse microbiome. Multilevel analyses, together with long exposure in the laboratory imitating conditions in a polluted environment, allow us to obtain a more complete picture of the impact of CAP on *D. magna*.

## Introduction

The recent extensive use of antibiotics in animal production, agriculture, and medicine leads to contamination of the environment (Manyi-Loh et al. 2018). Ingested medicines are removed from the body in unchanged form or as metabolites. One of the most important sources of pharmaceuticals, including antibiotics, entering surface waters is sewage from households and industrial sewage from pharmaceutical plants and hospitals (Dinh et al. 2017; Thai et al. 2018). For example, the concentration of the antibiotic chloramphenicol (CAP) in treated sewage in China reaches 1.73–2.43 µg L^−1^ (Peng et al. 2006). Conventional wastewater treatment methods, such as chlorination, are mainly used to neutralize microbiological contamination. Numerous studies show that traditional sewage treatment plants are not effective in eliminating antibiotics completely from sewage (Gulkowska et al. 2008; Hendricks and Pool 2012). The unintentional existence of pharmaceuticals in aquatic environments has harmful effects on aquatic organisms and the associated microbiome.

Chloramphenicol is commonly found in surface waters. CAP is a phenicol antibiotic with a broad bacteriostatic spectrum. Isolated in 1947, it was initially used to treat bacterial infections such as encephalitis, meningitis, and brain abscesses. However, later on, due to observed side effects, such as aplastic anemia (Rich et al. 1950) or gray baby syndrome (Sutherland 1959), CAP was classified as a very toxic substance. Currently, it is mainly used for the topical treatment of eye and skin infections. Based on data pointing to its genotoxicity (Hanekamp and Bast 2015; Rosenkranz 1988), CAP was classified as a probable human carcinogen by the International Agency for Research on Cancer (IARC 1990). In the European Union, there is a ban on the use of CAP in food production (EU Commission Regulation 2010). Unfortunately, research confirms the illegal use of the antibiotics in animal husbandry in Asia (Doğan et al. 2020; Hanekamp et al. 2003).

Antibiotics found in lakes are usually present in amounts ranging from 18.03 ng L^−1^ to 360 ng L^−1^ (Nguyen et al. 2022). Medicines usually act on those physiological pathways that are evolutionarily fixed in different taxa (Arnold et al. 2014). Pharmaceuticals are continuously delivered and accumulated in freshwaters, which results in chronic exposure of organisms inhabiting these environments. The presence of CAP has a negative impact on the “target” population of microorganisms in the aquatic environment, but it also affects non-target organisms, such as fito, zooplankton, and fish (Kumar et al. 2019).

*Daphnia* sp. can be perceived as such a non-target organism. As a filter feeder, *Daphnia* sp. performs an important function in freshwater ecosystems, occupying crucial place in the food chain of aquatic environments. It feeds mainly on organic matter suspended in water, and in turn, it constitutes food for plankton-eating fish and fry. *Daphnia magna* is one of the organisms most frequently used in toxicological studies, among other reasons, due to its high sensitivity to a wide range of chemical compounds. *D*. *magna* is a model organism in various fields of research (Harris et al. 2012; Seda and Petrusek 2011).

There are many studies in the literature on the acute toxicity of chloramphenicol to *Daphnia* sp. Studies indicate low acute toxicity of CAP to *D. magna* (e.g., 48-h EC50 effective concentration for immobilization was 372.4 mg L^−1^, and 96-h EC50 81.2 mg L^−1^) (Choi et al. 2008). For comparison, the LOEC, i.e., the lowest concentration at which significant adverse effects of CAP were observed, was 2.5 mg L^−1^ (Can et al. 2018). However, the low acute toxicity does not exclude the potential threat that may result from long-term exposure of *Daphnia* sp. to this antibiotic.

The ecological importance of this group of organisms is the main argument for undertaking research on the effect of chloramphenicol on *D. magna*. Since we are interested in the ecological impact of the pharmaceuticals as well as in the mechanism of their chemical toxicity at different biological levels, we analyzed the effect of long-time exposure (three generations) to CAP at environmental concentrations on *D. magna* proteome and life history. The *D. magna* microbiome was analyzed as well. To the best of our knowledge, this is the first such complex analysis performed on *D. magna*.

## Materials and methods

### Pharmaceuticals

Antibiotic Chloramphenicol (CAS: 56–75-7; purity greater than 98%) was purchased from Sigma‐Aldrich. A stock solution was prepared by suspending 1 mg of CAP in 1 mL of Milli-Q water and vortexing until completely dissolved. Then, a series of dilutions was performed to obtain a concentration of 1 µg mL^−1^. This solution was divided into 6 portions. In the following weeks, a new portion of the solution was used. Stock solutions were stored at 4 °C in the dark for the entire duration of the experiment (64 days). The working concentration was 32 ng L^−1^ of CAP, as is found in freshwater ecosystems (Yang et al. 2018) and was obtained by adding 32 µL of the 1 µg mL^−1^stock solution to 1 L of ADaM (see the “*Daphnia magna* clones” section).

### Daphnia magna *clones*

We used four *D. magna* clones, referred to as B, D, K, and P, taken from three separate ponds in the same region in order to obtain different genotypes while keeping environmental conditions relatively constant. The clones came from four reservoirs: (i) B was obtained from Germany (Binnensee, 53° 23′ 43.3″ N 12°37′ 56.0″ E); (ii) D was isolated from Domin pond (49°00′ 16.9″ N 14°26′ 21.3″ E) in the suburbs of Ceske Budejovice, Czech Republic; (iii) K and (iv) P originated from city park ponds in Warsaw, Poland (52°13′ 49.0″ N 21°01′ 43.0″ E) (52°12′ 42.0″ N 21°00′ 01.0″ E), respectively.

Experimental monocultures were established by isolating a single female of each clone from the clone library of the Department of Biochemistry and Microbiology (Warsaw University of Life Sciences, Poland). Second clutch neonates of each subsequent generations were used for further culturing and in the experiments. To standardize the pre-experimental conditions, the animals of each clone were cultured for at least three generations in the laboratory prior to the experiment. Both pre-experimental and experimental daphnids were cultured under constant conditions: in a climate cabinet (Plant Growth Chambers Sanyo MLR-350H) adjusted to 20 °C ± 0.5 °C, summer photoperiod (16L:8D; 0.30 ± 2 μmol s^−1^ m^−2^), and fed daily with green algae *Chlamydomonas reinhardtii* at a non-limiting growth concentration of 1 mg C_org_ L^−1^ (Lampert 1987). *D. magna* were cultured in “Aachener Daphnien Medium” (ADaM: CaCl_2_ • H_2_O (117.60 gL^−1^), NaHCO_3_ (25.2 gL^−1^), SeO_2_ (0.07 gL^−1^), and sea salt 0.3 gL^−1^; Klüttgen et al. 1994). The culture medium was changed every second day.

### Experimental setup

The experiment was performed with three subsequent generations of *D. magna*, lasting 64 days. The animals were treated with 32 ng L^−1^ CAP added to the medium. A medium devoid of pharmaceuticals served as a control. Although degradation of the pharmaceutical was unlikely, i.e., hydrolysis of 50% CAP in aqueous solution occurs after about 290 days (SIGMA product information), the experimental media were prepared with a fresh dose of CAP and changed every second day to avoid a potential decay of the antibiotic.

**Fig. 1:**
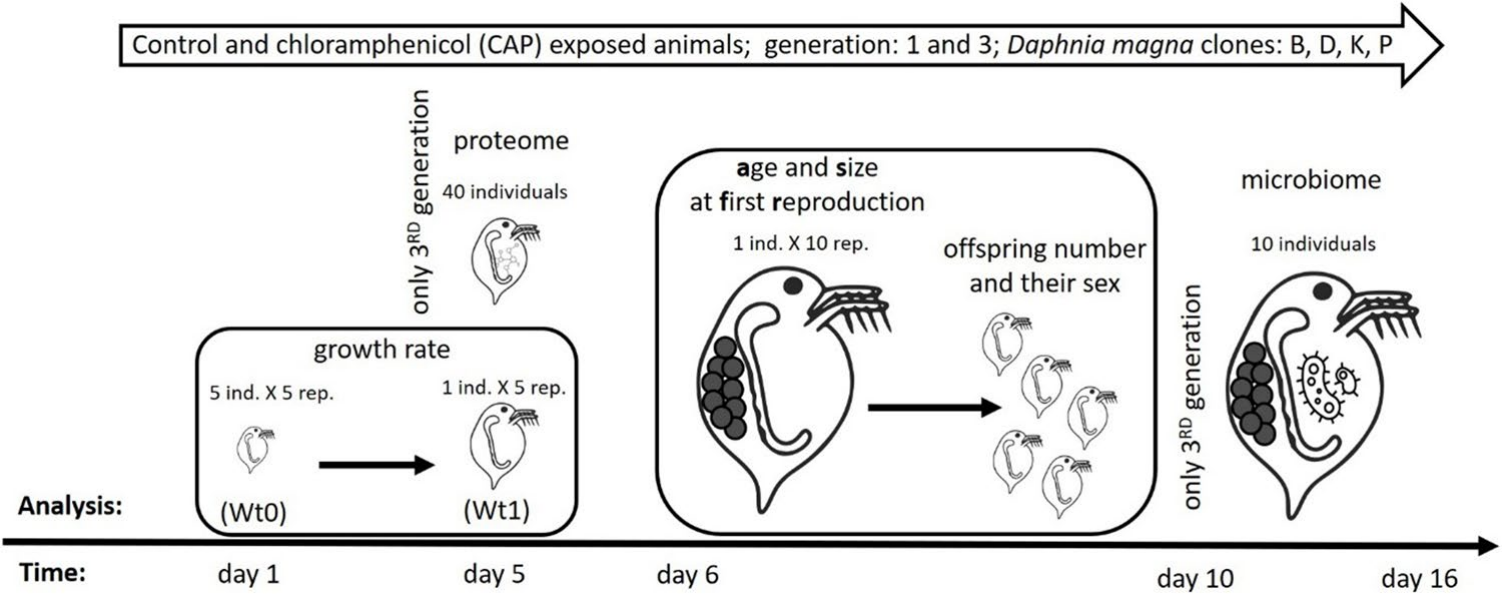
Experimental setup

Fifty animals were used for each treatment during the assessment of life history parameters (Fig. 1). Twenty-five of < 12-h-old daphnids and five of 5-day old animals were taken to determine body mass for further juvenile growth rate calculations. Reproduction-related parameters were recorded in the remaining 20 animals (see the “*Daphnia magna* life history parameters” section). Additionally, other 40 individuals from each clone were designated for proteome analysis. Daphnids used for the determination of life history parameters, as well as animals for proteome, were kept together in 4500 mL medium vol. (1 ind. per 100 mL). Proteome analyses were performed with third-generation, 5-day-old daphnids. The age corresponds to pre-adult instar grown at 20 °C. At this stage, females are sexually mature, but there were no eggs in the brood chamber. We performed proteome analyses at this life stage intentionally; the animals were old enough, so the effect of CAP should already be manifested. Additionally, because there was no egg in the brood chambers, the results of the proteome analyses were not biased by the developing embryos. Individuals for life history analysis were cultured until the second clutch offspring were released. As mentioned, second-clutch neonates constituted individuals of the next generation. All four clones were tested simultaneously. Additionally, at the end of the experiment, all animals (adults and neonates) from the third generation after the second reproduction were collected for microbiome analysis.

### *Daphnia magna* life history parameters

For each treatment and clone, five 5-day-old daphnids were dry-weighed (24 h at 60 °C) in order to calculate their individual juvenile somatic growth rate (Lampert and Trubetskova 1996). Ten remaining individuals from each clone were further observed to determine the age at first reproduction (AFR) and size at first reproduction (SFR), the number of neonates per female, and their sex (Fig. 1).

Dry mass increase was converted to juvenile somatic growth rates (gi) according to the formula gi = (ln[W_t1_] – ln[W_t0_]) × Δt-1 where (W_t0_) − weight of animals at the beginning of the experiment < 12-h-old, Wt1 5-day-old daphnid, Δt – time (in days) between the beginning of the experiment and the point of animal harvesting. Five-day-old *D. magna* were weighed separately to get dry body mass for W_t1_. The animals were weighed using an Orion Cohn C-35 Microbalance Thermo Scientific (exact within 0.1 µg) device.

To analyze life history parameters, we used a three-way ANOVA followed by a Tukey post hoc test (at *α* = 0.05) with Bonferroni correction to test the effect of the CAP on the *D. magna* life history parameters. Analyses were performed using Statistix 10.0 (Analytical Software, Tallahassee, USA). To evaluate the difference in the male share in *D. magna* populations, a chi-square independence test with Yates’ correction was performed in Statistica 13.

### Total protein isolation and mass spectrometry data analysis

After the indicated time points, 20 *D. magna* individuals, 5 days old, from all clones, were collected and placed in liquid nitrogen, with five replicates per treatment. Mass spectrometry analysis was performed in the Mass Spectrometry Laboratory (IBB PAS, Warsaw, Poland). The animals were homogenized in 95% trifluoroacetic acid (TFA) using a modified SPEED protocol (Doellinger et al. 2020). After 30 min of sonication in Biruptor (4 °C, 30 s ON/OFF, power High), the homogenate was neutralized by the addition of 2 M Tris and centrifuged (20,000 × g, 20 min, 4 °C). Fifty microliters of the resulting lysate was further two times diluted in 100 mM ammonium bicarbonate. Cysteines were reduced in 10 mM tris(2-carboxyethyl)phosphine (TCEP) followed by incubation with 20 mM s-methylmethanethiosulfonate (MMTS).

Proteins were digested overnight (16 h) with 3 µg of trypsin (Promega). Twenty microliters of each sample were measured on an Orbitrap Exploris 480 mass spectrometer (Thermo Scientific) coupled to an Evosep One chromatograph (Evosep Biognosys). Samples were loaded onto disposable Evotips C18 trap columns (Evosep Biosystems) according to the manufacturer’s protocol, with minor modifications as described previously (Jancewicz et al. 2021). Chromatography was carried out according to description published by Kamińska et al. 2023 with one exception, i.e., auto maximum inject time, and a scan range of 350 to 1400 m/z.

Raw data were processed with MaxQuant software (version 2.1.1) to obtain LFQ intensities and protein identifications using *Daphnia magna* reference proteome from UniProt (26,600 sequences) with contaminants included. Data investigation was completed in the Perseus suite (version 1.6.15). Data from the reversed database, contaminants, and proteins identified by the suite were removed. Data were log-transformed and a two-sample *t*-test was performed to estimate the statistical significance.

### Microbial community

Total DNA was extracted from 250 mg of *D. magna* per sample using a Fast DNA Spin Kit for Feces (MP Biomedicals) according to the manufacturer’s instructions. The DNA concentration was determined using the Qubit™ 2.0 Fluorometer (Invitrogen, Carlsbad, CA, USA).

The bacterial 16S rRNA genes were amplified from the extracted total DNA using the primers Bac341F (Muyzer et al. 1993) and Bac805R (Herlemann et al. 2011) at a concentration of 300 nM. PCR reaction was prepared using 0.5 U KAPA HiFi HotStart DNA polymerase (Kapa Biosystems, Inc, Wilmington, MA, USA). A template concentration of 10 ng and a total amount of 25 µL of reaction volume was used. PCR conditions comprised an initial denaturation at 95 °C for 3 min, followed by 24 cycles of denaturation (98 °C for 20 s), annealing (58 °C for 15 s), elongation (72 °C for 30 s), and a final extension step of 72 °C for 1 min. Six separate reactions were prepared, pooled, and purified using PCR/DNA Clean-Up Purification Kit (EURx, Gdansk, Poland). The sequencing libraries were constructed using MiSeq Reagent Kit v3 (Illumina). The libraries were later sequenced in 2 × 300 bp paired-end mode using a MiSeq instrument (Illumina) by Genomed SA (Poland).

Initial raw data quality control was checked using fastqc. Adapters removal and trimming was performed using a cutadapt tool (v 3.4) (Martin 2011). Trimmed sequences were imported into the Qiime2 software package (release 2021.11) for subsequent analysis (Bolyen et al. 2019). Read sequences were truncated (270/230 forward/reverse). Quality filtering, denoising, merging of paired-ended reads, and de novo chimera removal were performed using the DADA2 plugin (Callahan et al. 2016) in order to obtain Amplicon Sequence Variants (ASVs). Bacterial taxonomy was assigned for each of the ASVs using a pre-trained Naive Bayes classifier, based on the Silva 138 SSU database (Quast et al. 2012). The alpha diversity of the samples was assessed using a number of ASV, Faith phylogenetic diversity, Shannon, and Pielou’s Evenness indices. The paired sample *t*-test was used to assess differences between the analyzed groups (control vs chloramphenicol treatment). Raw sequencing data is available at the European Nucleotide Archive (ENA) under BioProject accession number PRJNA1027122 and sample accession numbers SAMN37779113- SAMN37779120.

## Results

### Life history parameters

**Fig. 2:**
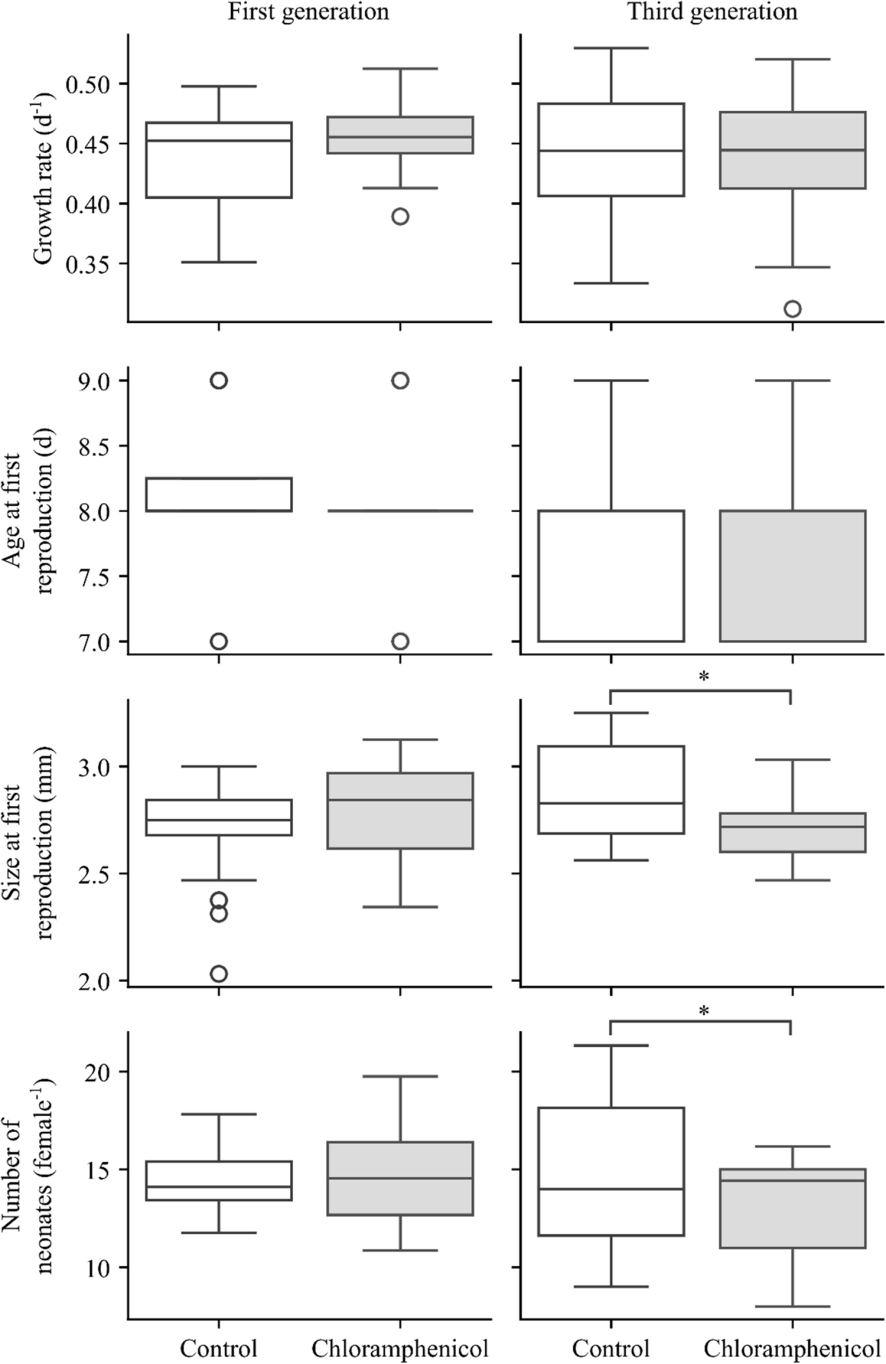
*Daphnia magna* (all clones) life history parameters. Boxplot showing the distribution of life history parameters across generation and treatment. The central line within each box represents the median, the edges of the box represent the first and third quartiles (Q1 and Q3), and the whiskers extend to the minimum and maximum values. Outliers are plotted as individual points beyond the whiskers as white circles. Star indicates significant differences among treatments (repeated-measure ANOVA, post hoc Tukey HSD)

Chloramphenicol exposure significantly affected *D. magna*’*s* size at first reproduction (SFR) and the number of neonates per female; however, it showed no significant effect on growth rate and age at first reproduction (AFR) (Table 1, Fig. 2). The post hoc analysis (Tukey HSD at *α* = 0.05) of the effect of pharmaceutical exposure revealed that CAP caused a smaller body size at first reproduction and a reduced number of born neonates per female when compared to the untreated control.

**Table 1:** Three-way ANOVA testing the effect of pharmaceutical treatment (no pharmaceutical, chloramphenicol), genotype (B, D, K, and P clone) and effect of generation (first, third) on *D. magna* life history parameters: growth rate, age, and size at first reproduction, number of neonates

Variable	Factor	df	*F*	*p*-value
**Growth rate**				
*n* = 5	Treatment (*T*)	1	0.48	0.4906
	Clone (*C*)	3	4.78	** < 0.01**
	Generation (*G*)	1	1.12	0.2945
	*T* × *C*	3	7.45	** < 0.001**
	*T* × *G*	1	2.05	0.1568
	*C* × *G*	3	8.96	** < 0.001**
	*T* × *C* × *G*	3	1.20	0.3166
	Error	64		
**Size at first reproduction**				
*n* = 10	Treatment (*T*)	1	4.06	** < 0.05**
	Clone (*C*)	3	23.27	** < 0.001**
	Generation (*G*)	1	3.04	0.0833
	*T* × *C*	3	1.74	0.1608
	*T* × *G*	1	20.09	** < 0.001**
	*C* × *G*	3	1.48	0.2232
	*T* × *C* × *G*	3	4.24	0.0067
	Error	138		
**Age at first reproduction**				
*n* = 10	Treatment (*T*)	1	2.97	0.0871
	Clone (*C*)	3	18.14	** < 0.001**
	Generation (*G*)	1	58.88	** < 0.001**
	*T* × *C*	3	0.61	0.6076
	*T* × *G*	1	0.02	0.8758
	*C* × *G*	3	9.31	** < 0.001**
	*T* × *C* × *G*	3	0.68	0.5666
	Error	144		
**Number of neonates per female**				
*n* = 10	Treatment (*T*)	1	13.63	** < 0.001**
	Clone (*C*)	3	175.39	** < 0.001**
	Generation (*G*)	1	12.50	** < 0.001**
	*T* × *C*	3	19.62	** < 0.001**
	*T* × *G*	1	31.32	** < 0.001**
	*C* × *G*	3	3.21	** < 0.05**
	*T* × *C* × *G*	3	29.94	** < 0.001**
	Error	135		

Bold entries indicate statistical significance

Further analysis of the treatment × generation interaction revealed that there was no significant difference in the SFR and the number of neonates between treatments in the first exposed generation. The observed adverse effect of CAP manifested only after chronic, i.e. three generations, exposure. On average, animals from four *D. magna* clones, after culturing for three generations in CAP, were smaller at first reproduction by 0.1687 mm in comparison to untreated animals. After chronic exposure to CAP, *D. magna* females had on average two individuals less in comparison to control organisms. An analysis of the treatment × generation × clone interaction revealed that in the first generation, there were no significant differences between treated clones in all life history parameters examined. Again, chronic CAP exposure led to a deleterious outcome in *D. magna*.

CAP-exposed animals from K and P clones in the third generation were smaller at first reproduction and had statistically less and statistically more neonates respectively in comparison to non-treated *D. magna*. Individuals from the B clone cultured with CAP produced fewer neonates in the third generation; however, their SFR did not differ from non-treated individuals. Interestingly, animals from the D clone showed no CAP effect in their life history parameters. However, among clone D animals exposed to CAP, we observed two *D. magna* females carrying ephippia.

**Fig. 3:**
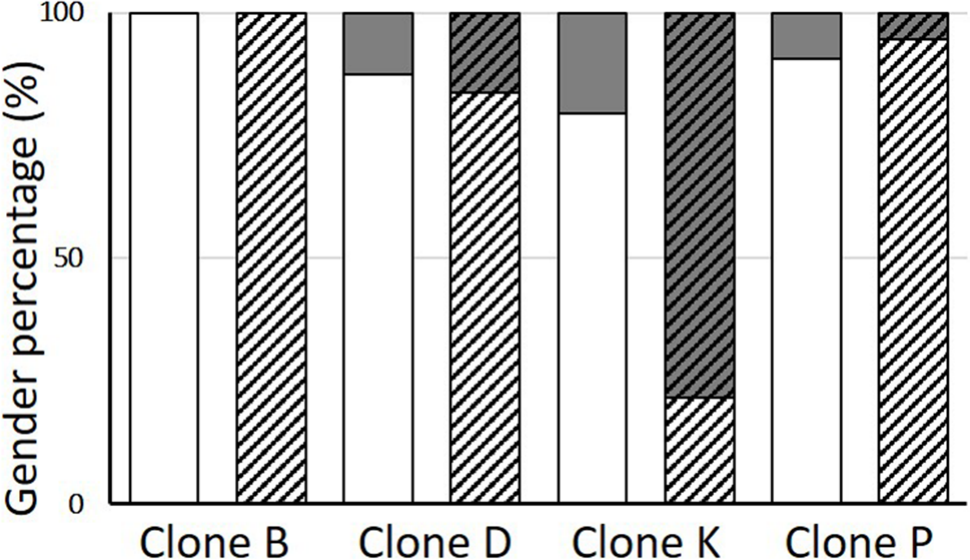
Gender percentage (females – white bar; males – grey bar) of *Daphnia magna* (B, D, K, and P clone) cultured without pharmaceutical (empty bars), in the presence of CAP (striped bars) after three generations

We found male production in three out of four tested clones (Fig. 3). In the B clone, no males were observed at all. In animals from the D and P clones, males appeared in the third generation, but the proportion of males to females was the same between individuals exposed to CAP and the control. Females from the K clone produced male offspring all the time as they originate from an unstable environment (Mikulski and Grzesiuk 2020). In the third generation, the sex ratio between the control *D. magna* and those exposed to pharmaceutical individuals was statistically significant (*X*^2^ = 19.61, *p* < 0.001). The *D. magna* not exposed to CAP produced 3.8 times more females than males. Females cultured in the presence of CAP produced over 3.5 times more males than females.

### The composition of *D. magna* proteins found by mass spectrometry after CAP treatment

Quantitative proteome analysis showed the presence of 820 proteins (proteins present in all replicates). The abundance of several proteins were observed, namely, the analysis revealed an upregulation of 17, and a downregulation of 34 proteins, in CAP-treated *D. magna* in comparison to untreated individuals.

Animals exposed to CAP produced more enzymes, such as: prolyl endopeptidase, aspartate aminotransferase, 4-hydroxyphenylpyruvate dioxygenase, deoxyhypusine hydroxylase, ubiquitin-conjugating enzyme E2 N, phenylalanine 4-monooxygenase, ATP synthase (delta subunit), thioredoxin domain-containing protein, and casein kinase II subunit alpha. They also produced structural proteins: cuticle protein, calcium-binding protein, gamma-adducin, tropomyosin-1, and vinculin, and other proteins: cytochrome b-c1 complex subunit Rieske (mitochondrial), aluminum tubes protein, putative basement membrane-specific heparan sulfate proteoglycan core protein.

Next, 40S ribosomal proteins (S3, S10, S13, S16, S20) and 60S ribosomal proteins (L7, L11, L12, L14, L27, L32) were expressed at a lower level in CAP-exposed *D. magna* than in unexposed. Furthermore, the 116 kDa U5 small nuclear ribonucleoprotein component was also downregulated. Another downregulated group of proteins under CAP conditions is related to metabolism and enzymes: chitin deacetylase, superoxide dismutase, UDP-glucose: glycoprotein glucosyltransferase, serine hydroxymethyltransferase, 26S protease regulatory subunit 6B, glutathione transferase. The remaining downregulated proteins were as follows: eukaryotic translation initiation factor 3 subunit K and D, two fragments of vitellogenin fused with superoxide dismutase, putative transcription factor A—mitochondrial, ATP synthase subunit e—mitochondrial, complement component 1 Q subcomponent-binding protein, bicoid stability factor, signal peptidase complex catalytic subunit SEC11, contactin associated protein-like 5–1, ATP-binding cassette sub-family F member, VWFD domain-containing protein, proteasome subunit beta type-1, putative chondroitin proteoglycan-2, DE-cadherin, and one uncharacterized protein (A0A164LPA0).

Also, our analysis showed the presence of an additional nine proteins, i.e. mitochondrial import receptor subunit, 5-hydroxyisourate hydrolase, FHA domain-containing protein, proteasome activator complex subunit, dusky-like protein, collagen alpha-1(I) chain, putative amphiphysin, reelin domain-containing protein, beta-lactamase 2-like protein and the absence of two, i.e., vitelline membrane outer layer protein 1 and protein PTCD3 (mitochondrial), in *D. magna* cultured with CAP in comparison to non-exposed individuals.

### Microbiome analysis

**Fig. 4:**
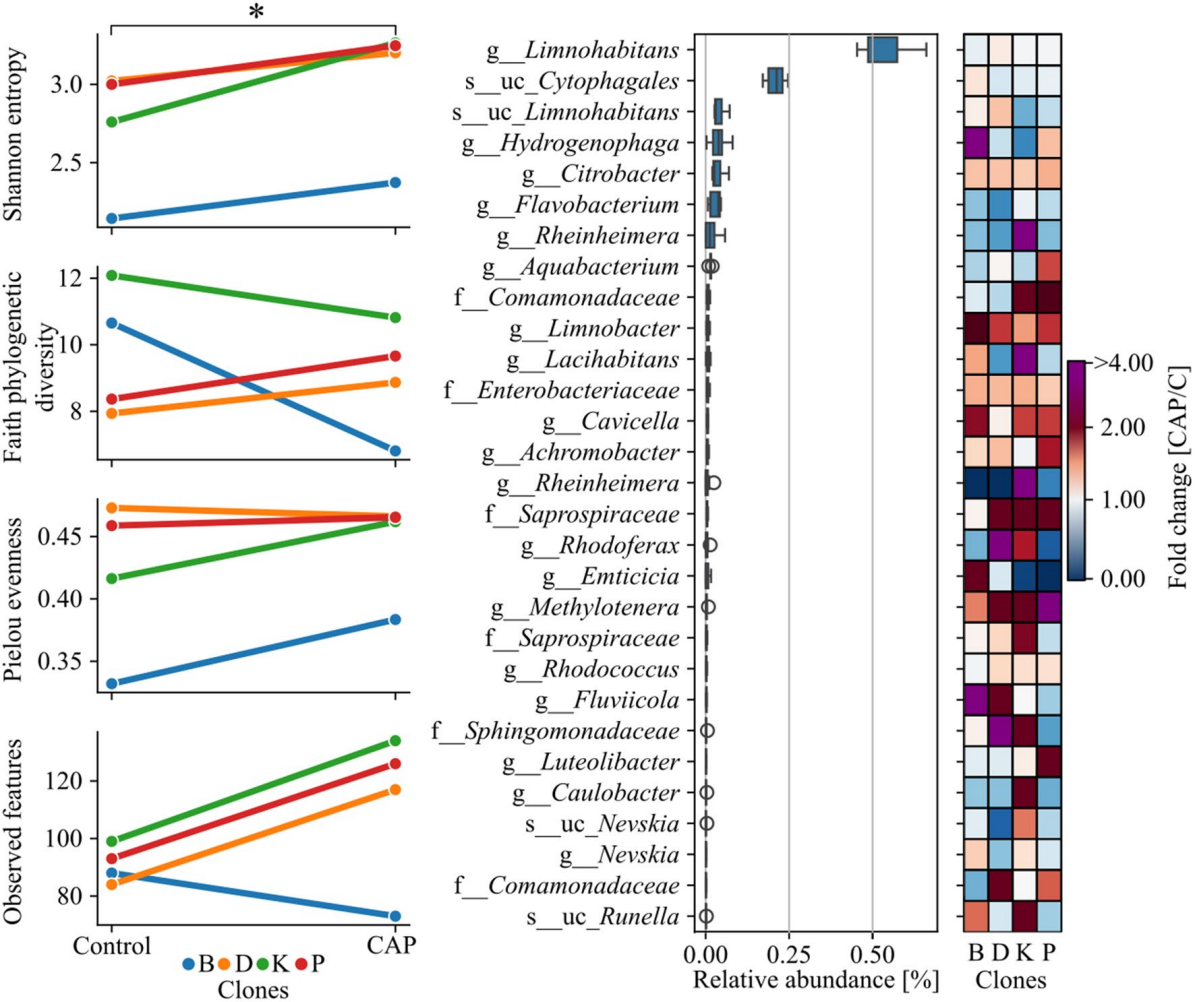
*Daphnia* microbiome analysis. A star symbol indicates a p-value less than 0.05. Bacterial taxa with a mean relative abundance of less than 0.1% in all samples were filtered out

A total of 1, 243, 542 paired-end reads were obtained from the DNA sequencing run. The average read count per sample was 155, 442 reads (min. 137, 907, max. 175, 906). During the quality control process, low-quality reads and nucleotides were removed, and the average number of reads per sample dropped to 928,308 (min. 102, 262, max. 131, 763). Rarefaction of the reads showed that all samples were saturated in terms of identified features. Results showed that the alpha diversity of the *D. magna* microbiome was relatively low in all analyzed clones. The average value of the Shannon entropy index was 2.87. The Faith phylogenetic diversity (PD) value varied between 12.08 and 6.81. The observed features, represented by ASVs, ranged from 73 to 134. The average Pielou evenness value was 0.43. A paired samples *T*-test showed a significant difference in the Shannon index between the control and the chloramphenicol-treated samples (Fig. 4). Overall, 12 different bacterial orders were identified. The highest relative abundance were observed for *Limnohabitas* sp. (average 52.26 ± 7.09%), an uncultured genus of *Cytophagales* (average 21.18 ± 2.57%), an uncultured *Limnohabitans* (4.18 ± 1.61%), *Hydrogenophaga* sp. (3.92 ± 2.27%), Citrobacter sp. (3.49 ± 1.67%) and Flavobacterium sp. (2.95 ± 1.45%) (Fig. 4). The results obtained showed that CAP treatment reduced the relative abundance of *Flavobacterium* in all clones. On the other hand, the results showed an increase in the relative abundance of the genus *Citrobacter* sp., *Limnobacter* sp., *Cevicella* sp., and *Methylothenera* sp., and undefined genera of the *Enterobacteriaceae* and *Saprospiraceae* families (Fig. 4).

## Discussion

The results obtained in the present study showed the effect of chronic (three generations) exposure of *D. magna* to chloramphenicol at a concentration detected in the environment. The antibiotic effects were observed at the individual, proteome, and microbiome levels.

### Life history parameters

When looking at life history parameters, we observed significant changes in the size at first reproduction and the number of neonates per female after three generations of exposure to CAP. Due to the lack of data on the effect of low concentrations of CAP on *D. magna*, the results obtained for other pharmaceuticals, where environmental concentrations of the tested compounds were used, served as a reference. Similar to the results presented here, exposure to environmental concentrations of metoprolol, ethinylestradiol, fluoxetine, and CAP affected the size at first reproduction (Dietrich et al. 2010; Grzesiuk et al. 2022). However, contrary to our study, in most ecotoxicological studies, the age at first reproduction is the life history parameter typically affected by pharmaceuticals. Exposure to environmental concentrations of carbamazepine, ethinylestradiol, metoprolol, ibuprofen, cisplatin, and cyclophosphamide changed the age at first reproduction (Dietrich et al. 2010; Grzesiuk et al. 2018, 2020; Lürling et al. 2006; Mielecki et al. 2023).

Study by Yuxuan et al. (2019) shows result after a 21-day exposure of *D. magna* to higher than environmental CAP concentrations, i.e., 0.05, 0.5, 1, 2.5, and 5 mg L^−1^. No significant effect of the pharmaceutical on body size was observed, while at a concentration of 0.5 mg L^−1^, the population growth rate decreased by 13.6% compared to the control. Moreover, the authors showed decrease in the number of offspring per female at CAP concentrations of 0.5 and 2.5 mg L^−1^. In the available literature, there is no data on the toxicity of CAP on the somatic growth rate of representatives of the *Daphnia* genus.

If we look only at the changes in life history parameters, we could consider that CAP is harmful to *D. magna* because in the third generation, the animals start reproducing at a smaller body size and have less neonates. However, the CAP effect depends on the clone origin. Animals from clones P and K do indeed reproduce at a smaller body size but have many offspring. This may be their strategy for dealing with CAP. Animals invest in offspring rather than somatic growth. In turn, animals from clone B have fewer offspring, and here, we can actually see the costs associated with being in the presence of the antibiotic.

### Life history parameters and proteome background

The proteome profile obtained in our experiments showed an upregulation of cuticula protein and a downregulation of chitin deacetylase. The latter protein is an enzyme found in the molting fluid, essential for apolysis and breakdown of the old cuticle and successful completion of the molting cycle (Soetaert et al. 2007). A downregulation of chitin deacetylase may account for the smaller body size at first reproduction of CAP-exposed individuals. This could also lead to an accumulation of cuticle protein that is not used during molting and growth. A similar reaction was observed in *D. magna* exposed to benzotriazoles. Two chitinases and one chitin deacetylase (cda3) coding genes were significantly upregulated and downregulated in response to this ubiquitous aquatic contaminant, respectively (Giraudo et al. 2017).

It is widely accepted that the amount of energy distributed toward growth and reproduction is reduced in response to chemical stress. To determine the effect of a chemical on the reproductive output of *D. magna*, changes in offspring number per female are taken into consideration, e.g., OECD Test No. 211. In our study, the number of neonates differed between exposed and non-exposed *D. magna* females. Despite vinculin upregulation in *D. magna* exposed to CAP, the modification of the offspring number differed between clones. Vinculin is a cytoskeletal protein that plays an important role in the regulation of focal adhesions and embryonic development (Humphries et al. 2007). We tested four *D. magna* clones, among which the individuals of two clones (B and K) showed a decrease in neonate number, females originating from one clone (P) increased offspring production, and *D. magna* from the D clone were not affected by CAP. The increase/decrease in the number of neonates influenced by the pharmaceutical depends on the clonal origin. Since *D. magna* shows high phenotypic plasticity (Tollrian and Dodson 1999) when testing more than one clone, different reactions are very often observed (e.g., Grzesiuk et al. 2018).

### *Daphnia* stress response

Changes in reproduction mode are a known strategy to avoid adverse environmental conditions, including exposure to toxins. When environmental conditions are favorable, crustaceans of the genus *D. magna* reproduce parthenogenetically. When conditions deteriorate, these organisms can switch to producing male and haploid sexual resting eggs (Hebert 1987). We can speculate that CAP-cultured daphnids prepare themselves for diapause since we observed male production (Fig. 3), although ephippia production was limited to two females in all tested clones during the whole experiment the protein profile justifies our speculation. The low level of vitellogenin fused with superoxide dismutase (SOD) in CAP-exposed individuals may be responsible for this situation. *Artemia* vitellogenesis in oviparity and ovoviviparity reproductive modes was studied by Chen co-workers (2011). Vitellogenin was found to own six copies of the consensus cleavage site, R-X-X-R, and a SOD-like domain at the N-terminus. It was found that the different profiles of the oviparous and ovoviviparous pathways only involve SOD-containing subunits. Researchers deduced that these essentials play a key role in the production of encysted diapausing embryos during long-term cell cycle stoppage. Previously, SOD-like domains for vitellogenesis proteins have been described in *D. magna* (Grzesiuk et al. 2018; Kato et al. 2004; Tokishita et al. 2006).

Antibiotics, such as CAP, actively take part in different physiochemical and biochemical changes. Creating free radicals, such as reactive oxygen species (ROS), has been one of such specific chemical reactions (Teo et al. 1986; Matés and Sánchez-Jiménez 1999). In the present study individuals cultured with CAP for three generations show elevated levels of Cytochrome b-c1 complex subunit Rieske. Cytochrome bc1, also known as mitochondrial complex III, is considered to be one of the significant ROS producers (Pagacz et al. 2021). One of the consequences of excessive ROS formation is lipid peroxidation (Benzie 1996). It is assumed that lipid peroxidation may increase lipid degradation and, in this way, reduce lipid storage. As a result, decreased lipid-based ATP production coming from reduced fatty acid oxidation may result in reproductive malfunction (Houten et al. 2016). This case was observed in the present study. Increased production of thioredoxin domain-containing seems to confirm our presumption. This protein is involved in redox reactions and can be induced by oxidative stress. Increased production of proteins such as mitochondrial import receptor subunit, 5-hydroxyisourate hydrolase, FHA domain-containing protein, and proteasome activator complex subunit may be a reaction to oxidative stress, which also may be caused by the action of free radicals or toxic chemicals.

Induction of antioxidant enzymes superoxide dismutase (SOD) is a part of defence mechanisms against ROS in freshwater organisms such as *D. magna*. The enzymatic activities of ROS-induced SOD have been extensively verified as a biomarker for the assessment of stressors present in the environment (Tang et al. 2011; Yu et al. 2009). In the present study, SOD was downregulated in *D. magna* individuals in the presence of CAP. A similar response was observed when *D. magna* were exposed to hydroxymethylfurfural (Swart et al. 2019) or cyclophosphamide (Mielecki et al. 2023).

ROS-inducing compounds contribute to structural changes in mitochondria (Wakabayashi and Karbowski 2001), causing their dysfunction through changing the mitochondrial membrane and impairing oxidative phosphorylation (Zorov et al. 2006). This is why we detected changes concerning mitochondrial proteins in the proteome profile: complement component 1 Q subcomponent-binding protein, putative transcription factor A – mitochondrial, ATP synthase subunit e – mitochondrial, putative transcription factor A – mitochondrial, mitochondrial import receptor subunit and increased production of enzymes: 4-hydroxyphenylpyruvate dioxygenase, deoxyhypusine hydroxylase, phenylalanine 4-monooxygenase.

The observations and analyses listed above indicate that CAP is a stress factor for *D. magna*. Females cultured with CAP showed different levels of proteins related to stress response in comparison to non-treated individuals. We detected an up-regulation of ubiquitin-conjugating enzyme E2 and calcium-binding protein. E2 enzymes perform the second step in the ubiquitination reaction that targets a protein for degradation via proteasomes. The ubiquitin–proteasome pathway is a well-studied mechanism that plays an important role in regulating protein homeostasis and trafficking. Ubiquitination regulates various cellular processes, including immune response, angiogenesis, cell proliferation, apoptosis, and DNA repair (Ciechanover 1998; Myung et al. 2001). In *D. magna*, transcriptional responses after 8-day exposure to gamma radiation also show ubiquitin-conjugating enzyme E2 upregulation (Song et al. 2020).

The most common calcium-binding protein is calmodulin, involved in signal transduction as well as the response to stress (Laneve et al. 2008; Lee et al. 2021; Kuro-o et al. 1997; Olejnik et al. 2018). In our previous study, we observed calmodulin upregulation and downregulation in response to cyclophosphamide and cisplatin treatment, respectively (Mielecki et al. 2023). Also, Song and co-workers (2020) observed changes in the levels of calmodulin in *D. magna* under different doses of gamma radiation.

Furthermore, we noted a downregulation of glutathione transferase and 26S protease regulatory subunit 6B. Glutathione transferase is a part of the immune and stress response. It plays a role in detoxification originating from xenobiotics (Hayes et al. 2005; Josephy 2010). As with the proteins listed above, changes in the regulation of this enzyme were observed when *D. magna* was exposed to gamma radiation (Song et al. 2020). 26S protease regulatory subunit is a multiprotein complex involved in the ATP-dependent degradation of ubiquitinated proteins. This complex plays a key role in the maintenance of protein homeostasis by removing misfolded or damaged proteins impairing cellular functions, and by removing proteins whose functions are no longer required. Therefore, the proteasome participates in numerous cellular processes, including cell cycle progression, apoptosis, and DNA repair.

Yuxuan et al. (2019) showed changes in levels of biochemical stress markers after 24 h of *D. magna* exposure. 0.001 mg L^−1^ of CAP significantly increased the malonaldehyde and reduced glutathione levels. On the other hand, catalase (CAT) a common antioxidant enzyme was downregulated. These results confirm ours—CAP causes oxidative stress response in *D. magna*.

### Other effects

CAP is an inhibitor of the 50S ribosomal subunit. This antibiotic prevents the formation of new peptide bonds on the ribosome, inhibiting the biosynthesis of bacterial proteins (Scholar 2007). Interestingly, we observed a downregulation of several ribosomal proteins from both subunits (40S and 60S) in *D. magna* individuals exposed to CAP. Multigenerational exposure of *Daphnia* to tetracycline also shows changes in ribosomal proteins from subunit 40 and 60 subunit (Kim et al. 2012) Moreover, we did not detect PTCD3 protein in the proteome profile of animals cultured with CAP in comparison to non-treated individuals. PTCD3, a member of the pentatricopeptide repeat domain protein family, is a component of the small mitoribosomal subunit (Amunts et al. 2015; Greber et al. 2015). This is another peptide showing changes concerning mitochondria.

### *Daphnia* microbiome

Chloramphenicol shows a broad spectrum of activity useful for the treatment of gram-positive and gram-negative bacteria infections. This antibiotic is a stable compound and is difficult to remove from water environments. It was shown that CAP from the farm and aquatic animal industry could be released into the environment through feces. The concentration of released CAP in the water bodies varies between 2.0 ng L^−1^ (Tong et al. 2009) and approx. 0.5 µg L^−1^ (Hirsch et al. 1999). According to the EUCAST, the minimal inhibitory concentration (MIC) inhibiting microorganism growth observed for CAP is 1 mg L^−1^.

The most dominant genus observed in all four clones was *Limnohabitans* sp. This is in agreement with previous studies that showed the *Burkholderiaceae* family as the most abundant in *D. magna* individuals across genotype, population, and temperature level (Frankel-Bricker et al. 2020). Other research has shown that bacteria of the R-BT linage have a crucial role in freshwater bacterioplankton communities (Kasalický et al. 2013). It was demonstrated that *Limnohabitans* sp. inhabits a broad range of freshwater niches and can constitute up to 30% of free-living bacteria in freshwater ecosystems (Zwart et al. 2002). The second most dominant genus identified in all variants was the uncultured representative of *Chitinophagales.* Previous studies showed that using a metagenomic approach, *Limnohabitans* and *Chitinophagales* were commonly identified as bins within the *D. magna* microbiota (Cooper and Cressler 2020). The results obtained showed that Shannon diversity increased over the chloramphenicol treatments (Fig. 4). This was caused by the increased number of unique ASVs in clones D, K, and P and increased evenness in the case of clone B. Interestingly, a relatively high proportion of the *Citrobacter* spp. and *Enterobacteriace*ae families were identified within the analyzed samples. It was shown that the relative abundance of those taxa increased after exposure to CAP. Previous studies have shown that daphnids have the potential to carry the tetracycline-resistant gene *tet(A)* (Eckert et al. 2016; Perera et al. 2022).

## Conclusions

Pharmaceuticals polluting freshwater ecosystems can modify organisms living there, even at low concentrations, given their constant presence in the environment. We are the first to expose *D. magna* for three generations to environmental concentrations of CAP and to use multilevel analyses to detect the pharmaceutical effect. The reaction of *D. magna* to the presence of CAP indicates their high stress. Our results show that CAP changes *D. magna*’s life history parameters, proteome, and microbiome. The individuals were smaller and on average produced fewer offspring. The differences in *D. magna*’s life history after CAP treatment were in accordance with proteome changes. Interestingly, the CAP-exposed *D. magna* proteome profile confirms that CAP, as a ROS-inducing compound, contributes to structural changes in mitochondria. CAP-exposed animals have a more diverse microbiome in comparison to non-treated daphnids. It is necessary to further extend the exposure of *D. magna* to the pharmaceutical to check whether and how the animal’s condition will stabilize. Specifically, will there be more life history disorders, and will the microbiome continue to diversify under the influence of CAP? Moreover, increasing the number of clones representing different enterotypes would allow for checking whether the antibiotic effect is enterotype dependent. Metagenome analyses would also complete the picture. Last but not least, studying the proteome profile of animals carrying eggs or embryos may show the CAP effect from a different perspective.

## Data Availability

Data available upon request.
